# Aortic Incompetence and Its Lessons

**Published:** 1910-07-30

**Authors:** J. Mitchell Bruce

**Affiliations:** Consulting Physician to the Hospital.


### Hospital Clinics.

### AORTIC INCOMPETENCE AND ITS LESSONS.

By J. MITCHELL BRUCE, M.D., F.R.C.P., Consulting Physician to the Hospital.

(A Clinical Lecture delivered at the Hospital for Consumption and Diseases of the Chest, Brompton, on Wednesday, June 1, 1910.)

Ladies and Gentlemen,—My junior colleagues—if I may so call them, for I am no longer myself on the active staff of the Brompton Hospital—have been good enough to ask me to come here to-day and give a clinical lecture on some subject which I am permitted to select for myself. I have taken as the subject of the lecture a topic which in itself is of great interest—namely, aortic incompetence—and, in the second place, is peculiarly fruitful of instruction both in respect of the mechanics of valvular disease of the heart and associated affections of the vessels, and in regard to the basis of the prognosis and treatment of valvular disease of the heart. In other words, it furnishes us with a number of lessons which those of you who are qualified will not resent my putting before you, although you are practitioners yourselves, because it is as students that I once more choose to address you.

I have to make a selection of the points which I wish to dwell upon, and I want to take the subject up in rather a special order. And, first, I would mention simply the cardinal physical signs of incompetence of the aortic valves. They are very simple. There are the auscultatory signs; secondly, the signs significant of hypertrophy and dilatation of the heart; and, thirdly, the signs associated with the arterial system—that is, with the pulse and certain other tracts of the arterial system ahead of the heart. I will take them in that special order.

### The Murmurs.

I select one of the patients here as an instance of the disease, and begin as a student often begins when he is learning his medicine—with the stethoscope. What is it that we find? We find a diastolic murmur, which we call an aortic diastolic, a characteristic murmur. That murmur has many characters which I need not dwell upon to-day, because I am sure you are familiar with them; I mean with respect to its loudness and where it is formed, and with respect to its conduction and its changes under different circumstances. I only remind you that it is formed in the situation of the aortic valve. It has to be traced by what we call the method of conduction. The aortic systolic murmur is traced up along the direction of the blood-current into the vessels. You know the mitral systolic murmur is formed in very much the same situation; there is only a fraction of an inch between the two places where they are formed. Now this aortic diastolic murmur is conducted right away from the place where it is formed in the direction of the line of the left ventricle, so that it is often heard best along a line running obliquely downwards across the left cartilages. In other instances it is best heard at the end of the sternum, a natural sounding-board, *i.e.* at the ensiform cartilage. And no doubt most of you know that this aortic diastolic murmur is sometimes not audible at the base at all, but can only be caught at the lower end of the sternum or somewhere at the apex. Let us now interpret its significance. What does it mean? All of you who understand the significance of the second sound of the heart must conclude that such a diastolic murmur is significant of an incompetence of the valve. You have, instead of a second sound due to the shutting or tightening of the aortic cusps, a blowing murmur, sometimes of very considerable length, which is due to the reflux of the current of the blood out of the aortic arch into the left ventricle; and there is no other explanation. And it is interesting to be reminded that the physiology of the second sound of the heart was established in this way by experiment, that is, by destroying the aortic valves in an animal and so producing a diastolic murmur, and it was worked out by one of the first physicians of this hospital. Before that time the significance of the second sound was not known.

### Cardiac Enlargement.

The next cardinal sign of aortic incompetence is the evidence of enlargement of the heart. If we take one of those patients, you will find the precordial impulse is remarkably increased in two respects. Instead of the apex beating in the fifth interspace inside the nipple line, it presents an extensive precordial impulse extending even into the seventh intercostal space. So we have here the second observation, from which we may learn a very easy lesson. It can only mean this—that the heart is much larger than it was before. You may say it is displaced, but it is simply that there is a larger surface of heart beating against the chest-wall. What does it mean? It means that the heart is dilated. How does it come to be dilated? It is more capacious and is holding more blood. But where does this blood come from? With our stethoscopes we have just found that the blood is regurgitating out of the aortic arch back into the left ventricle; and the ventricle is holding so much more blood than it ought to, because it is being filled from the left auricle and from the aortic arch at the same time. It is dilatation of a cardiac cavity by over-filling.

### The Cardiac Impulse.

Next I would point out to you that this impulse is not only very extensive, but is very powerful. What is the meaning of this great increase in force? It can only mean that the muscular wall of the heart is contracting with much more vigour than normal, and this can only mean one thing if it is permanent—namely, that the heart is hypertrophied. How has the hypertrophy arisen? There is no difficulty about that. The blood has regurgitated into the left ventricle and has over-filled it, and now at each systole this left ventricular muscle has to throw out an increased amount of blood. So we have the double parietal change which is usual in valvular disease—dilatation with hypertrophy. Here we have in a simple way a demonstration of what is known as compensation of a valvular lesion by enlargement of the heart. I want you to note once more how it occurs. In this lesion compensation is produced, first, by accommodating the amount of blood which is regurgitated out of the aorta. What would happen if the ventricle did not dilate? Obviously in a few seconds the heart would be choked if it did not dilate and make room for the blood which has regurgitated. So we are entitled to say that dilatation by overfilling is a compensatory, useful provision against regurgitation through the aortic valves. In other words, our hearts are highly elastic bags, which will dilate and accommodate any moderately increased quantity of blood which may flow into them. The second element, the hypertrophy, is, as we know, a most precious provision of another order. In its origin it is referable to a provision which we possess physiologically—that if we exercise our muscles sufficiently, with all the circumstances favourable to nutrition, and without excessive or unreasonable demand on them, the muscles will act more and will grow. In this way we have dilatation and hypertrophy going on side by side; and the result is compensation. I might have percussed out the precordial dulness and shown you that it agrees with what you feel with your hands, but that is not necessary. Another point which I might mention before leaving the physical signs is that if we study not only the second sound (or its absence, that is to say, the regurgitant murmur) but also the auscultatory signs at the mitral orifice, we occasionally hear Flint's murmur. If you listen in these cases you will find that the first sound in the mitral area is accentuated, a little like the first sound in mitral obstruction: it is of a sharp snapping quality. It appears that the current which is regurgitating from the aorta into the left ventricle crosses the anterior cusp of the mitral valve and prevents that valve opening freely when the auricle contracts—a condition of virtual obstruction.

### Corrigan's Water-hammer Pulse.

The third cardinal evidence of aortic incompetence is the condition of the arterial system ahead of the lesion. In the first place, there is pulsation in the second right interspace, visible and palpable, possibly with some thrill. Secondly, there is pulsation in the vessels of the neck. And, thirdly, there is what is familiar to you all, Corrigan's pulse. Let us consider what the significance of all these may be, and draw a few more lessons from it. The abnormal aortic pulsation presents two characters: first, there is increased force; and, secondly, the pulsation is extensive towards the right, beyond the limits of the aortic arch. That means dilatation: the aortic arch in aortic incompetence is dilated. This might at first sight appear a curious result to some of you. If the blood regurgitates out of the arch, the arch should get smaller. But the arch is dilated in systole, for this great heart throws at each systolic output not only its normal amount of blood but also that amount which it had received from the aorta immediately before. That dilates the vessel, and you will always find the aortic arch dilated in aortic incompetence, because it has to accommodate an increased charge of blood thrown into it with great force.

Now look at the next point where the vessels come into view—namely, above the clavicles. There you find great pulsation with each systolic movement of the left ventricle. The subclavians are overfilled, stretched, strained in each systolic movement. We follow the arteries down and finally come to the radial, where we find Corrigan's pulse, so named after Sir Dominic Corrigan, the distinguished physician of Dublin. If you study Corrigan's pulse you will find it is, as a rule, regular. It may be frequent or infrequent, according to the condition of the patient. It is rather large or of good volume. It is extraordinarily soft in cardiac diastolic time—that is to say, between the beats—in fact it collapses; and if the hand is raised over the head it still further empties itself in diastolic time, and I have come across a case in which the pulse failed to reach the finger when the hand was held high over the head. Further, the stroke is a sharp and powerful one. Let us interpret those features. A good volume of blood comes into the vessel. Next, there is collapse. What is the meaning of that? We revert to the observation we have made—the aortic diastolic murmur, signifying regurgitation of blood: the pulse wave fails all along from the radial back into the heart. Now we come to the other features of Corrigan's pulse, and they are, first, that it is visible; secondly, that it is tortuous; thirdly, that it is locomotive—you can see it moving at each systolic output. What is the meaning of those three characters? We saw that the ventricle suddenly throws a large quantity of blood into the arterial tree; we have seen its effect in the chest and in the neck; we have a large, sharp, sudden pulse: no wonder it is visible. Why is it tortuous? For the explanation of this we go back to the observations which we have made at the base of the heart and in the neck. I showed you that when the ventricle contracts, and throws this large volume of blood into the aorta, the aortic arch is dilated. The same thing goes on throughout the whole arterial tree, which is stretched in aortic incompetence. And when a vessel is stretched in this way by internal pressure, it enlarges in two directions: it becomes a wider vessel than before because it is stretched, and it also becomes longer. The result is that the whole arterial system is too long for the limbs; and so to accommodate the lengthened vessels they become tortuous. The more tortuous and the larger and thicker the vessel, obviously the greater has been the disturbance of circulation at the heart. You can use the radial artery and its degree of tortuosity as a true index or measure of what has been going on in the heart. This is a very good instance of how much we can learn by feeling the pulse, not only the healthy pulse but the pulse when it is disturbed or unnatural. Take one of the ordinary instruments for registering the pressure of the pulse, and you find a remarkable difference between the mean pressure, where the movement of the column of mercury is most marked, and the systolic pressure where you obliterate the pulse altogether.

Now, what is this state of the circulation that we have found? We have the arterial tree suddenly filled with an abnormal amount of blood in ventricular systole, and as instantly emptied of it again. We have seen that the effect is manifested in the pulse, in the neck, and in the chest itself. But these are only the accessible vessels, which we get at and see and feel. What about the other arteries? What is the effect of this great gush of blood which is thrown in at one moment and allowed to escape at the next moment? What is the effect on the coronaries? They must be more or less like the radials—tortuous, thickened, and lengthened; and whatever the amount of compensation may be, it cannot be called a satisfactory circulation—there must be an insufficient amount of blood in the arterial and capillary systems. The coronaries and their capillaries are in a precarious condition, and compensation itself is precarious because the hypertrophied left ventricle is dependent for its function upon the nutrition of its walls, and that nutrition depends on the circulation in the coronaries and their capillaries. In aortic incompetence there is a very insecure condition of compensation.

But in addition to the heart there are all the other viscera. And as the brain is at the highest point of the circulation, that also is in a precarious condition as to nutrition. You find these patients complaining not only of palpitation, pain, and discomfort about the heart, but also of faintness, vertigo, and general weakness—of what we should call symptoms of anæmia. This is in marked distinction from the dyspnœa and other evidences of pulmonary choking which we meet with in mitral lesions.

This is all that I have to say about the simple mechanics of aortic incompetence.

And now some of you, at least those who have not studied the condition fully, may be prepared to say: "I think I can now diagnose a case of aortic incompetence: I should hear the murmur, make out for myself the size of the heart, and note Corrigan's pulse, and the other points you have told me of; and I could offer a prognosis. And, knowing the condition of the coronaries and of the brain, I think I could order treatment, planning that on what you have told me about the state of the heart." Let me correct you. On what I have told you you cannot reach prognosis or treatment. After we have passed the stage of the student and the young practitioner, in which we are so interested in physical signs, listening to these remarkable murmurs, making our sphygmographic tracings, and getting our blood-pressures, we find that something more is necessary before we can prognose or treat a case of aortic incompetence. We find a patient whom we treated for acute rheumatism with endocarditis, and who has an aortic incompetence murmur as a result of his endocarditis, coming back at the end of ten years—perhaps with a bad toe or a bad finger. We examine his heart, and when we find he still has his aortic murmur we are surprised to find that he is well. But before we have been very long in practice we come upon another case of aortic incompetence, and, to our surprise, instead of the man living on comfortably, everything has gone wrong: there has either been sudden syncope with its ordinary unhappy effects, or the patient rapidly falls into a condition of cardiac failure, as it is called, with dropsy. Then we ask ourselves, Why should one be different from another? We discover that there is something more than the mere mechanics of the condition to be discussed. The patient I mentioned with rheumatic aortic incompetence is one who had endocarditis and who is left with a simple scar, or pucker, or irregularity, on one or more of his aortic valves, which had healed, and his compensation is established; and though he is somewhat disabled he continues well. The other case did not have rheumatism, but he had syphilis, or he strained one of his valves (and he may have been, very likely was, syphilitic first); or he has arterial degeneration, possibly along with chronic Bright's disease and high tension; or he may have had gout with arterial sclerosis of the terminal arteries or of his aortic arch, and there is an incompetent valve behind the high blood-pressure. This condition is a different one to deal with. There is no old healed lesion, but a progressive degeneration. So the practitioner finds that in addition to all these beautiful physical problems, which are necessary and instructive to solve, he has also to consider the pathological problem proper. This is what I am afraid is often forgotten in making a complete diagnosis in aortic incompetence. How can you prognose without considering the pathological element? One man lives; the other man dies. And how different the treatment must be in the two cases! The one man, whose condition is due to rheumatism, does not require treatment; the other man requires large doses of iodide of potassium. Another man, with high blood-pressure, has to be treated accordingly; to be taken off his meat and off all stimulants, to be set at rest, sent away from business, and treated in a way which is calculated to reduce his arterial tension. An old man who has had rheumatic trouble may simply be treated on ordinary principles, but the old man who has a degenerated heart and who is getting worse under some of the conditions that I have mentioned has to be considered from a totally different point of view.

So far I have demonstrated to you patients who are the subjects of aortic regurgitation with compensation of the lesion effected by dilatation and by hypertrophy. Unhappily there is another phase of this disease—that of cardiac failure where compensation is lost. A few days ago I saw in consultation a man suffering from præcordial distress and restlessness, with dropsy of the legs and scanty, albuminous urine, who had happened to consult me many years before, but whose case I had altogether forgotten. The face was pale, with a tint of jaundice; the pulse frequent, small, feeble, and irregular. I found the præcordial impulse weak and diffused below and outside the left nipple line; the præcordial dulness more extensive than normal in all directions; and small, weak, irregular cardiac sounds, with an occasional murmur in systole over the mitral area. Over both pulmonary bases there were crepitations, and friction-sound over the right mammary region. The liver was large and tender; and there were doubtful signs of slight ascites. Obviously the condition was one of cardiac failure, and we diagnosed cardiac strain because of the history of acute development after severe exertion.

On my return home I referred to my old notes of this case, and found to my surprise that it was one not of cardiac strain but of aortic incompetence, probably syphilitic in origin. So completely had the classical characters of this lesion disappeared that it was unrecognisable as such. Of course the diagnosis was not only wrong, but unreasonably arrived at. We ought to have remembered that cardiac failure with the signs I have mentioned might be the last phase of any of the different forms of valvular disease. I have related this case to you to impress on you the appreciation of the complete change of condition and of symptoms and signs when the great powerful left ventricle of aortic incompetence fails. Fails to do what? Fails to complete its rhythmic systole—to discharge its proper *quantum* of blood, because of some weakening of the myocardium due to coronary disease or other event or influence. Then the ventricle becomes over-distended, its walls stretched, its cavity further dilated, for in this phase also more than the normal amount of blood has to be accommodated within it. But note: not because it has been over-filled, but because *it has not been sufficiently emptied.* The dilatation of failure is dilatation with residual blood, like the dilatation of an over-distended bladder. It is significant of feebleness; and it essentially negatives the possibility of hypertrophy, unless the pressure is relieved backwards by the mitral yielding or by the intake from the veins being reduced, or unless the force of the parietal contraction is increased artificially with better blood or with suitable drugs. When this is accomplished the mitral murmur, significant of "safety," leakage disappears, and the pressure, again rising in the aorta, restores the aortic regurgitant murmur. You will now appreciate, I trust, the difficulties that attend the diagnosis of aortic incompetence in its final stage. The prognosis and treatment of such a case is correspondingly difficult; but these are subjects beyond the scope of my lecture to-day.

